# THSD7A-associated membranous nephropathy involves both complement-mediated and autonomous podocyte injury

**DOI:** 10.3389/fphar.2024.1430451

**Published:** 2024-07-17

**Authors:** Jing Liu, Deepak Malhotra, Yan Ge, William Gunning, Lance Dworkin, Rujun Gong

**Affiliations:** ^1^ Division of Nephrology, Department of Medicine, Toledo, OH, United States; ^2^ Department of Pathology, The University of Toledo College of Medicine, Toledo, OH, United States

**Keywords:** Thsd7a, membranous nephropathy, complement, podocyte, cytoskeleton, apoptosis, mouse model

## Abstract

Membranous nephropathy (MN) continues to be a leading cause of nephrotic syndrome in non-diabetic adults. As a unique subtype in the serology-based classification of MN, thrombospondin type 1 domain containing 7A (THSD7A)-associated MN has attracted increasing interest, because, unlike other autoantigens, THSD7A is also expressed in preclinical species, facilitating the study of its role in MN. A heterologous mouse model of THSD7A-associated MN was previously established using a proprietary in-house antibody that was unfortunately not available to the research community. Here, we developed a mouse model of THSD7A-associated MN by administering a commercially available antibody targeting the most N-terminal part of THSD7A. Our model was characterized by heavy proteinuria and pathological features of human MN without sex differences. Complement depletion with cobra venom factor only partially attenuated proteinuria and glomerular injury in this model, entailing that complement-independent pathomechanisms also contribute. Consistently, *in vitro* in primary podocytes, exposure to the anti-THSD7A antibody caused evident podocytopathic changes, including disruption of actin cytoskeleton integrity, podocyte hypermobility, oxidative stress, and apoptotic cell death. These signs of podocytopathy were preserved, albeit to a lesser extent, after complement inactivation, indicating autonomous podocyte injury. Furthermore, as the first FDA-approved treatment for primary MN, adrenocorticotropic hormone therapy with repository corticotropin injection (Purified Cortrophin Gel^®^) appeared to be beneficial and significantly attenuated proteinuria and glomerular injury, suggesting that this model may be useful for developing novel treatments or understanding the pathogenesis of MN. Collectively, our model, based on the use of a commercially available anti-THSD7A antibody, will be an important tool for MN research.

## Introduction

Membranous nephropathy (MN) is one of the most common causes of nephrotic syndrome and chronic kidney disease in the adult population (Cattran, 2005). As a pathologically defined disease of renal glomeruli, MN is histologically characterized by an apparent thickening of the glomerular capillary walls, which results from accumulation of electron-dense deposits on the outer aspect of the glomerular basement membrane (GBM) under electron microscopy (EM). Aside from complement components, the subepithelial deposits in glomeruli consist of immunoglobulin G (IgG) against the relevant antigens, including podocyte autoantigens in primary MN (pMN), or exogenous antigens or neo-epitopes planted *in situ* in the subepithelial space in secondary MN (Couser, 2017; Ronco et al., 2021). Being a typical immune complex-mediated autoimmune disease, pMN centrally involves immune dysregulation that leads to the loss of self-immune tolerance to podocyte autoantigens and the production of circulating nephritogenic antibodies (Hoxha, Reinhard and Stahl, 2022).

While the mechanism underlying the perturbations in B cell tolerance to podocyte antigens in pMN is unknown, the discovery of target antigens involved in the pathogenesis of pMN has allowed for a more precise molecular diagnosis (Behnert et al., 2014; Du et al., 2014). Moreover, the ability to monitor circulating autoantibodies has added a new dimension to immunosuppressant therapy monitoring and disease prognostication (Ruggenenti et al., 2015; Ramachandran et al., 2018). In the past 15 years, a number of autoantigens have been discovered in patients with pMN. Approximately up to 80% of pMN patients have found to be associated with autoantibodies against the glomerular phospholipase A2 receptor 1 (PLA2R1) antigen (Beck et al., 2009). However, the exact role of PLA2R1 autoantibodies in the pathogenesis of pMN has not been fully elucidated. This delay is largely due to the lack of animal models for mechanistic translational studies, as PLA2R1 is not expressed in many experimental animals, including rodents (Rinschen et al., 2018). In addition to PLA2R1, other autoantigens have been identified and implicated in pMN, including thrombospondin type 1 domain-containing 7A (THSD7A) (Tomas et al., 2014), semaphorin 3b (Sethi et al., 2020b), protocadherin 7 (Sethi et al., 2021) and neural epidermal growth factor-like 1 (Sethi et al., 2020a). Among these, THSD7A is expressed in podocytes in both human and other species (Godel, Grahammer and Huber, 2015; Meyer-Schwesinger, Lambeau and Stahl, 2015). THSD7A autoantibodies prepared from pMN patients could specifically bind to THSD7A on mouse podocytes, resulting in proteinuria and a histopathological pattern that is typical of human MN (Tomas et al., 2016). Furthermore, an in-house prepared anti-mouse THSD7A antibody was able to induce a heterologous mouse model of MN that was highly reminiscent of human THSD7A-associated MN with respect to proteinuria and histopathologic features (Tomas et al., 2017). However, the anti-THSD7A antibody used in this study was generated by the investigators using an in-house method and is not available to the research community. To facilitate the understanding of the pathogenesis of THSD7A-associated MN and to explore novel therapies, it is imperative to develop a pragmatic mouse model that is easy and practical to establish by using commercially available antibodies against THSD7A.

Despite progress in the identification of pMN-associated autoantigens, the pathogenic mechanisms responsible for glomerular destruction in pMN remain controversial and uncertain. As a typical immune complex-mediated glomerulopathy, MN is considered an archetypal complement-dependent form of glomerular disease, as evidenced by the concomitant deposition of complement components in the glomeruli of MN specimens (Salant, Belok, Madaio and Couser, 1980; Baker et al., 1989; Schubart et al., 2019). Therefore, complement has traditionally been considered as a key driver of glomerular injury in pMN. However, this dogma has been challenged by recent evidence. First, most autoantibodies detected in pMN and deposited in diseased renal glomeruli, such as anti-THSD7A, are IgG4, which does not effectively bind complements and is unable to activate the complement pathway (Beck, 2017). In addition, in rats with passive Heymann nephritis (PHN), a typical model of MN, glomerular injury and proteinuria still occurred in the absence of complement membrane attack complex (MAC) formation (Spicer et al., 2007). These findings suggest that complement-independent glomerular injury may play a key role in pMN.

The present study aimed to test this hypothesis *in vivo* in a heterologous mouse model of THSD7A-associated MN established by using a commercial antibody against THSD7A, and *in vitro* in a cell culture model of podocytopathy relevant to THSD7A-associated MN. As a further test of the utility of this mouse model of THSD7A-associated MN, we assessed the efficacy of Repository Corticotropin Injection (RCI; Purified Cortrophin^®^ Gel, ANI Pharmaceuticals, Inc., Baudette, MN, United States), which is an FDA approved treatment indicated to induce a diuresis or a remission of proteinuria in the nephrotic syndrome without uremia of the idiopathic type or that due to lupus erythematosus.

## Materials and methods

### Animal study

Animal experiments were performed in the Department of Laboratory Animal Resources (DLAR) at the University of Toledo College of Medicine after approval by the Institutional Animal Care and Use Committee of the University of Toledo (400176), and they conform to the US Department of Agriculture regulations and the National Institutes of Health guidelines for the humane care and use of laboratory animals.

All mice were housed in the DLAR at the University of Toledo with free access to water and regular chow diet. To induce the mouse model of MN, wild-type (WT) C57BL/6 mice of either sex at the age of 8–12 weeks received a single tail vein injection of a rabbit anti-mouse THSD7A antibody (0.5, 1.0, 2.5 or 5.0 μL/g body *wt*; LifeSpan BioSciences, Inc., Shirley, MA, United States) or the same doses of rabbit nonimmune control IgG. Animals were followed for indicated days and euthanized by inhalation of CO2. To deplete complements, mice were injected intraperitoneally with 10 μg of cobra venom factor (CVF; Complement Technology, Inc., Tyler, TX, United States) 1 day before and 1 day after injection of the anti-THSD7A antibody, and the control group received an equal volume of phosphate buffered saline (PBS). To test the efficacy of RCI, mice received a subcutaneous injection of RCI (30, 60 or 100 IU/kg body *wt*; Purified Cortrophin^®^ Gel, ANI Pharmaceuticals, Inc., Baudette, MN, United States) every other day starting 1 day before injection of the anti-THSD7A antibody, while the control group received injection with an equal volume of vehicle gel. Mice were euthanized on day 3, and urine and kidney specimens were collected.

### Kidney specimen preparation and glomerular isolation

Glomeruli were isolated by using a conventional magnetic separation technique as described previously (Li, Ge, Dworkin, Peng and Gong, 2016). Mice were euthanized and immediately perfused via cannulation of the left ventricular or the abdominal aorta. To flush out any remaining blood, both kidneys were perfused first with ice-cold sterile PBS until the kidneys had blanched. Then the left kidney was either resected and prepared for further histological examinations or subjected to subsequent perfusion together with the right kidney using PBS containing iron oxide particles. In brief, the infusion system was replaced by a syringe pump and the right kidney was perfused again with 10 mL of PBS containing magnetic iron oxide particles (Sigma-Aldrich, St. Louis, MO), followed by resection of the right kidney for isolation of glomeruli. Renal cortices were minced into 1 μm^3^ pieces and digested in collagenase A at 37°C for 30 min with gentle shaking. Tissues were pressed gently through a 100-μm cell strainer (BD Falcon, Bedford, MA, United States), and glomeruli containing iron oxide particles were gathered using a magnetic particle concentrator and prepared for subsequent immunoblot analysis or primary culture of podocytes.

### Urine protein analyses

Protein compositions of urine samples were determined by SDS-PAGE followed by coomassie brilliant blue (Sigma-Aldrich) staining. Urinary albumin concentrations were analyzed using a mouse albumin enzyme-linked immunosorbent assay (ELISA) quantitation kit (Bethyl Laboratories Inc, Montgomery, Texas, United States), and urine creatinine concentrations were measured by a creatinine assay kit (BioAssay Systems, Hayward, CA, United States). The severity of proteinuria was estimated by urinary albumin to creatinine ratios (uACR).

### Kidney histology

Formalin-fixed paraffin-embedded kidney specimens were prepared into 3-μm-thick sections and subjected to periodic acid-Schiff (PAS) staining. The slides were visualized using a Nikon Eclipse Ni-U microscope (Nikon Instruments, Melville, NY, United States) in a blinded manner by an investigator.

### Transmission electron microscopy

Kidney cortical tissues were cut into small pieces (1 mm^3^), fixed with 2.5% glutaraldehyde (Electron Microscopy Sciences, Hatfield, PA, United States), and processed using standard procedures. The grid sections were visualized using a Talos L120C transmission electron microscope (Thermo Fisher Scientific, Waltham, MA, United States). To determine the average foot process width, specimens from 3 animals per group were analyzed with a minimum of 30 measurements per animal. The linear size of the foot processes that touched the GBM was determined using ImageJ (version 1.52a; National Institutes of Health, Bethesda, MD), as previously described (Koop et al., 2003).

### Primary culture of podocytes

Primary podocytes were prepared from the isolated glomeruli as described before (Xu, Ge, Liu and Gong, 2014). Purified glomeruli were plated on Petri dishes coated with collagen I and incubated at 37°C in RPMI 1640 medium (Life Technologies, Grand Island, NY, United States) supplemented with 10% fetal bovine serum (FBS; Life Technologies), 100 μg/mL streptomycin, and 100 U/mL penicillin (Life Technologies) in an incubator with 5% CO2. Podocytes at passage 1 or 2 expressed multiple podocyte-specific markers and were used in subsequent experiments. In some experiments, FBS was heated at 56°C for 30 min prior to addition to the culture medium to inactivate the complements.

### Culture of immortalized mouse podocytes

Conditionally immortalized mouse podocytes in culture were a gift from Dr. Stuart Shankland (University of Washington, Seattle, WA, United States). Cells were cultured in RPMI 1640 medium (Life Technologies) supplemented with 10% FBS (Life Technologies), 0.075% sodium bicarbonate (Sigma-Aldrich), 0.075% sodium pyruvate (Sigma-Aldrich), 100 U/mL of penicillin, and 100 μg/mL of streptomycin (Life Technologies) in a humidified incubator with 5% CO2. Cells were cultured at 33°C with 50 U/mL of recombinant mouse interferon-γ (Millipore, Billerica, MA, United States) on collagen-coated Petri dishes. Cells were then transferred to 37°C incubators without interferon-γ to induce differentiation. Differentiated cells were collected for immunoblot analysis or prepared for immunocytochemistry staining.

### Cellular viability assay

Cellular viability was measured using the 3-(4,5-dimethylthiazol-2-yl)-2,5-diphenyl-2H-tetrazolium bromide (MTT) assay as described previously (Ghasemi, Turnbull, Sebastian and Kempson, 2021). After incubating for 1 h with differing doses of anti-THSD7A antibody, MTT (Sigma-Aldrich) at a final concentration of 0.5 mg/mL was added to each well, and the plate was further incubated at 37°C for 4 h for the signal to develop. Finally, dimethyl sulfoxide (Sigma-Aldrich) was added to each well. The plate was placed on a shaker in order to dissolve the dye. The optical density (OD) was measured at 570 nm using the Cytation 5 multi-mode microplate reader (BioTek Instruments, Winooski, VT, United States).

### Wound healing assay

Confluent monolayers of primary podocyte cultures were scraped with a 10 μL pipette after treatment with the anti-THSD7A antibody. Phase contrast micrographs were obtained at 0 and 24 h after scratching by using an EVOS FL microscope (Thermo Fisher Scientific). The wound areas at 0 and 24 h were analyzed using ImageJ, and data were expressed as the percentage of wound closure, as previously reported (Bao, Ge, Peng and Gong, 2015).

### Detection of reactive oxygen species (ROS) generation by fluorescence

The production of ROS in cultured primary podocytes was evaluated by detecting the fluorescence intensity of 2′,7′-dichlorofluorescein-diacetate (Zhu et al., 2011) (DCFDA, Invitrogen, IL, United States). Briefly, podocytes were grown on a 24-well plate and treated with rabbit anti-THSD7A antibody for 1 h. After treatment, cells were incubated with 5 μM DCFDA for 20 min at 37°C and then counterstained with Hoechst 33342 (Thermo Fisher). Cells were then visualized using a fluorescence microscope (EVOS FL, Thermo Fisher Scientific) and the fluorescence intensity was measured with a Cytation 5 multi-mode microplate reader (BioTek Instruments, Winooski, VT, United States) at an excitation wavelength of 488 nm and an emission wavelength of 528 nm.

### Terminal deoxynucleotidyl transferase (TdT) dUTP nick-end labeling (TUNEL)

Apoptotic cell death in cell cultures was detected by using the TUNEL kit (Promega, Madison, WI, United States) according to the manufacturer’s instructions. Following TUNEL staining, cells were counterstained with propidium iodide (PI; Abcam, Boston, MA, United States) and visualized by using the Nikon Eclipse Ni-U microscope (Nikon, Tokyo, Japan).

### Immunofluorescence staining

Cryosections of kidney specimens or cultured cells were fixed, permeabilized, and stained with antibodies against indicated molecules followed by staining with a secondary antibody conjugated with Alexa Fluor 488 or 594, or stained with rhodamine-conjugated phalloidin (Invitrogen) followed by counterstaining with 4,6-diamidino-2-phenylindole (DAPI, Abcam). Images were captured using the Nikon Eclipse Ni-U microscope (Nikon, Tokyo, Japan) and the Leica TCS SP5 multiphoton laser scanning confocal microscope (Leica Microsystems Inc., Buffalo Grove, IL, United States). Mean fluorescence intensity was analyzed with ImageJ software.

### Western immunoblot analysis

Isolated glomeruli were homogenized and cells were lysed in radio-immunoprecipitation assay buffer supplemented with a protease inhibitor cocktail (Thermo Fisher Scientific). Samples were subjected to western immunoblot analysis as described before (Fang et al., 2022). Anti-synaptopodin (SYNPO), podocin, desmin and glyceraldehyde-3-phosphate dehydrogenase (GAPDH) antibodies were purchased from Santa Cruz Biochemistry. The early growth response 1 (EGR1) and β-tubulin antibodies were acquired from Cell Signaling Technology. For immunoblot analysis, bands were scanned, and the integrated pixel density was determined using the ImageJ.

### Statistical analyses

Data were presented as mean ± SD, or otherwise as indicated. All data were statistically analyzed using SPSS 16.0 software. Data from two groups were compared by *t*-test. One-way ANOVA was used to compare continuous variables among groups when appropriate. For analyses of uACR over time, data was analyzed by repeated-measures ANOVA, followed by the *post hoc* Scheffé test. All *in vitro* studies were repeated at least 3 times. *p* < 0.05 was considered statistically significant.

## Results

### THSD7A expression is enriched in glomerular podocytes in mouse kidney and is recognized by a commercially available antibody that targets the N-terminal domain of mouse THSD7A

**FIGURE 1:**
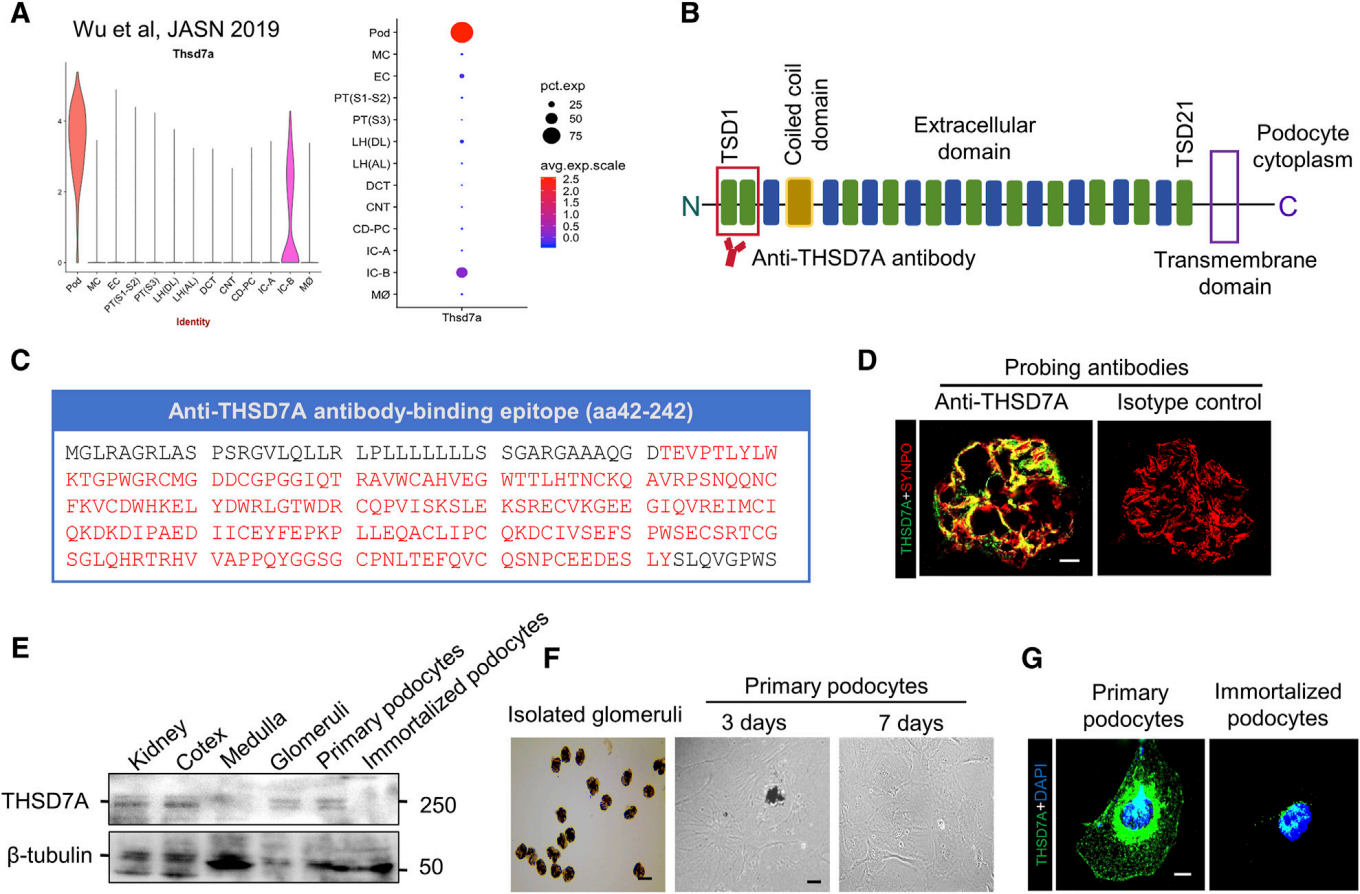
THSD7A is predominantly expressed in the glomeruli and is enriched in podocytes in mouse kidney. **(A)** A *post hoc* analysis was performed on the single nucleus RNA sequencing (snRNAseq) transcriptome of mouse kidney based on the Wu Healthy Mouse Dataset that is publicly-available from Kidney Interactive Transcriptomics [https://humphreyslab.com/SingleCell/], and evaluated the mRNA expression of THSD7A in various kidney cells, revealing a highly enriched expression pattern in glomerular podocytes. Pod, podocyte; MC, mesangial cell; EC, endothelial cell; PT (S1-S2), proximal tubular (Segment 1–2); PT (S3), proximal tubular (Segment 3); LH (DL), loop of henle (descending limb); LH (AL), loop of henle (ascending limb); DCT, distal convoluted tubule; CNT, connecting tubule; CD-PC, collecting duct principal cell; IC-A, alpha intercalated cell; IC-B, beta intercalated cell; M∅, macrophage. **(B)** Schematic view of the structure of THSD7A, which is a type 1 transmembrane protein with a large extracellular N-terminal region comprising 21 thrombospondin type 1 domains (TSDs) and a coiled coil domain in the extracellular (Seifert et al., 2018). The predominant target of autoantibodies in THSD7A-associated pMN exists in two domains in the most N-terminal region highlighted by red lines. **(C)** Antigen epitopes in THSD7A (amino acid 42–242) targeted by the commercial antibody used in this study are highlighted in red. **(D)** Representative micrographs showing fluorescent immunohistochemistry co-staining of THSD7A and SYNPO in mouse glomeruli. As isotype controls, nonimmune IgG was used as primary antibody and no specific staining was noted. Scale bar = 10 μm. **(E)** Representative immunoblot analysis of the indicated specimens for THSD7A and β-tubulin. **(F)** Representative micrographs showing isolated mouse glomeruli and primary culture of glomerular podocytes. Scale bar = 50 μm. **(G)** Representative micrographs showing fluorescent immunocytochemistry staining of THSD7A in primarily cultured mouse podocytes and immortalized mouse podocytes. Scale bar = 7.5 μm.

To determine the patterns of THSD7A expression in mouse kidney, a *post hoc* analysis of the single nucleus RNA sequencing transcriptome of mouse kidney was performed based on the Wu Healthy Mouse Dataset (Wu, Kirita, Donnelly and Humphreys, 2019) that is publicly-available from Kidney Interactive Transcriptomics (https://humphreyslab.com/SingleCell/). Shown in Figure 1A, the mRNA expression of THSD7A is predominantly detected in glomeruli and highly enriched in podocytes in mouse kidney (Figure 1A). Recent evidence suggests that the most N-terminal region of THSD7A is the predominant epitope for nephritogenic autoantibodies in THSD7A-associated MN in humans (Figure 1B) (Seifert et al., 2018). To validate this finding, cryosections of mouse kidney specimens were processed for fluorescence immunohistochemistry staining by using a commercially available rabbit anti-mouse THSD7A antibody directed against the most N-terminal domain of mouse THSD7A, spanning amino acid residues 42 to 242 (Figure 1C). Immunohistochemistry demonstrated a podocyte-specific staining pattern of THSD7A that appeared to colocalize with staining for the podocyte marker protein SYNPO (Figure 1D). In contrast, no specific staining was noted in staining with nonimmune isotype control. Consistently, immunoblot analysis indicated that THSD7A expression was detected in mouse whole kidneys, renal cortices and isolated glomeruli, but absent from renal medullary specimens (Figure 1E). In addition, THSD7A expression was probed abundantly in primary mouse podocytes, but barely in immortalized mouse podocytes, as shown by immunoblot analysis and fluorescent immunocytochemistry staining (Figures 1E–G). Therefore, primary cultures of mouse glomerular podocytes were used for subsequent *in vitro* models of podocytopathy related to THSD7A-associated MN.

### Anti-THSD7A antibody elicits podocytopathic changes in primary mouse podocytes

**FIGURE 2:**
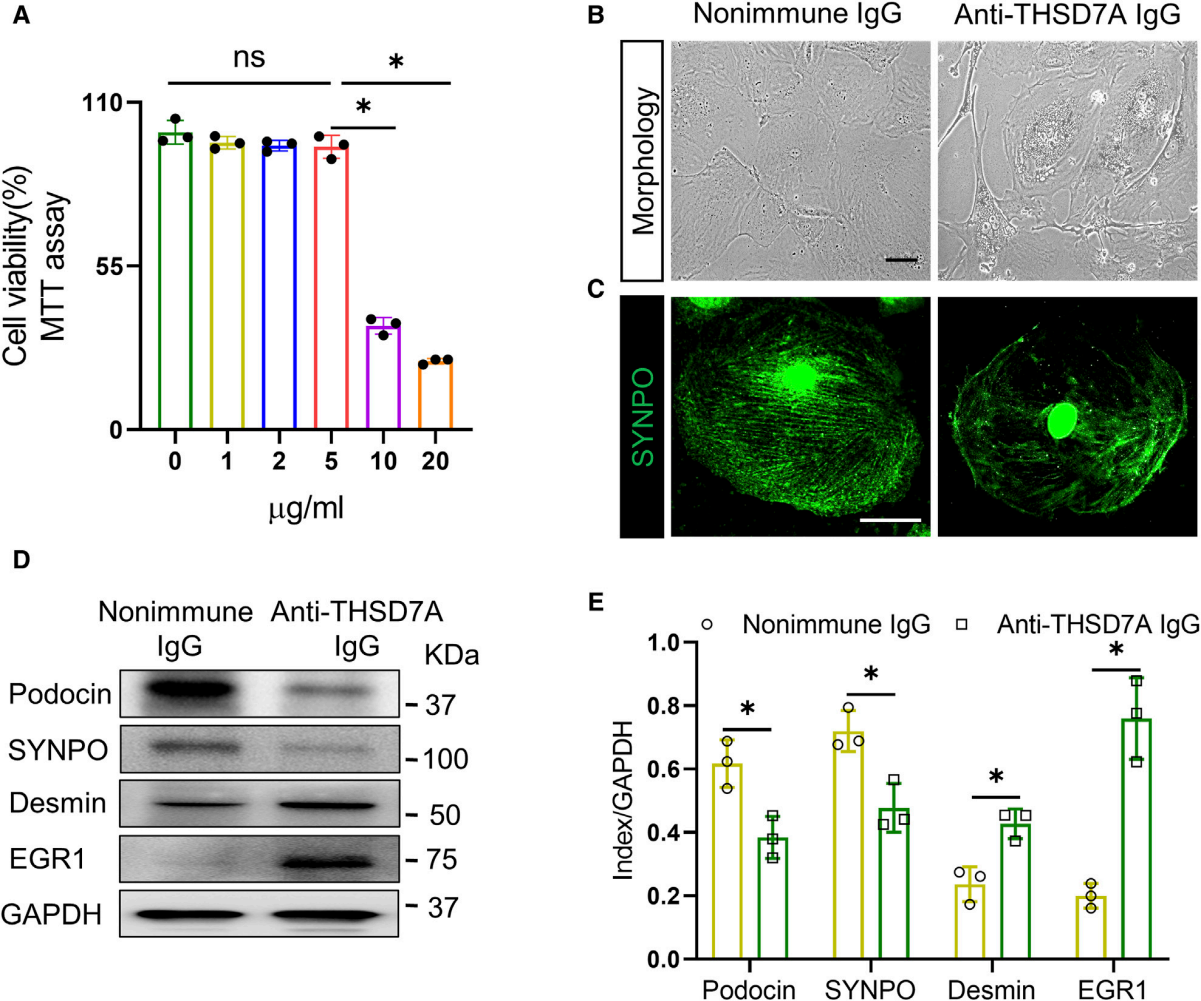
Anti-THSD7A antibody elicits podocyte injury. Primary cultures of mouse podocytes were exposed to the anti-THSD7A antibody at a concentration of 5 μg/mL, or as otherwise indicated, in the presence of normal culture medium containing 10% FBS for 1 h. **(A)** Cellular viability was assessed by a tetrazolium-based colorimetric MTT assay. *p < 0.05 (n = 3); ns, not significant. **(B–C)** Representative micrographs showing **(B)** distortion of cellular shape under phase contrast microscopy and **(C)** disruption of SYNPO expression (green) shown by fluorescence immunocytochemistry staining (Scale bar = 25 μm) in podocytes treated by anti-THSD7A antibody. Nonimmune IgG treatment served as controls. **(D)** Representative immunoblot analysis of cell lysates for indicated molecules. **(E)** Quantification of the immunoblot data by densitometric analysis. *p < 0.05 *versus* nonimmune IgG treatment for each molecule (n = 3).

To recapitulate podocytopathy in THSD7A-associated MN, primary mouse podocytes were treated with the anti-THSD7A antibody at varying concentrations for 1 h in the presence of normal culture medium containing 10% FBS. Cellular viability, based on the MTT assay, revealed that 5 μg/mL was the highest dose of anti-THSD7A antibody that barely affected cellular viability (Figure 2A). Therefore, 5 μg/mL was chosen as the optimal concentration of this antibody for the subsequent experiments. At this dose, the anti-THSD7A antibody, as compared with the nonimmune IgG, evidently elicited signs of podocytopathy, characterized by podocyte morphologic changes of podocytes from large, flat, and arborized cell shapes to cellular shrinkage, reduced arborization, aster-like cell shapes, and vacuole formation under phase contrast microscopy (Figure 2B). These changes were associated with disruption of the staining patterns of the podocyte homeostatic marker SYNPO based on fluorescence staining (Figure 2C). The morphologic findings were further corroborated by immunoblot analysis coupled with densitometric analysis, which demonstrated an apparent loss of expression of podocyte canonical marker proteins such as podocin and SYNPO, but a significant increase in expression of podocyte injury markers including desmin and EGR1 (Figures 2D, E). Taken together, these data demonstrate that the anti-THSD7A antibody possesses podocytopathic activities.

### Anti-THSD7A antibody induces podocyte hypermotility, oxidative stress and apoptosis in primary mouse podocytes

**FIGURE 3:**
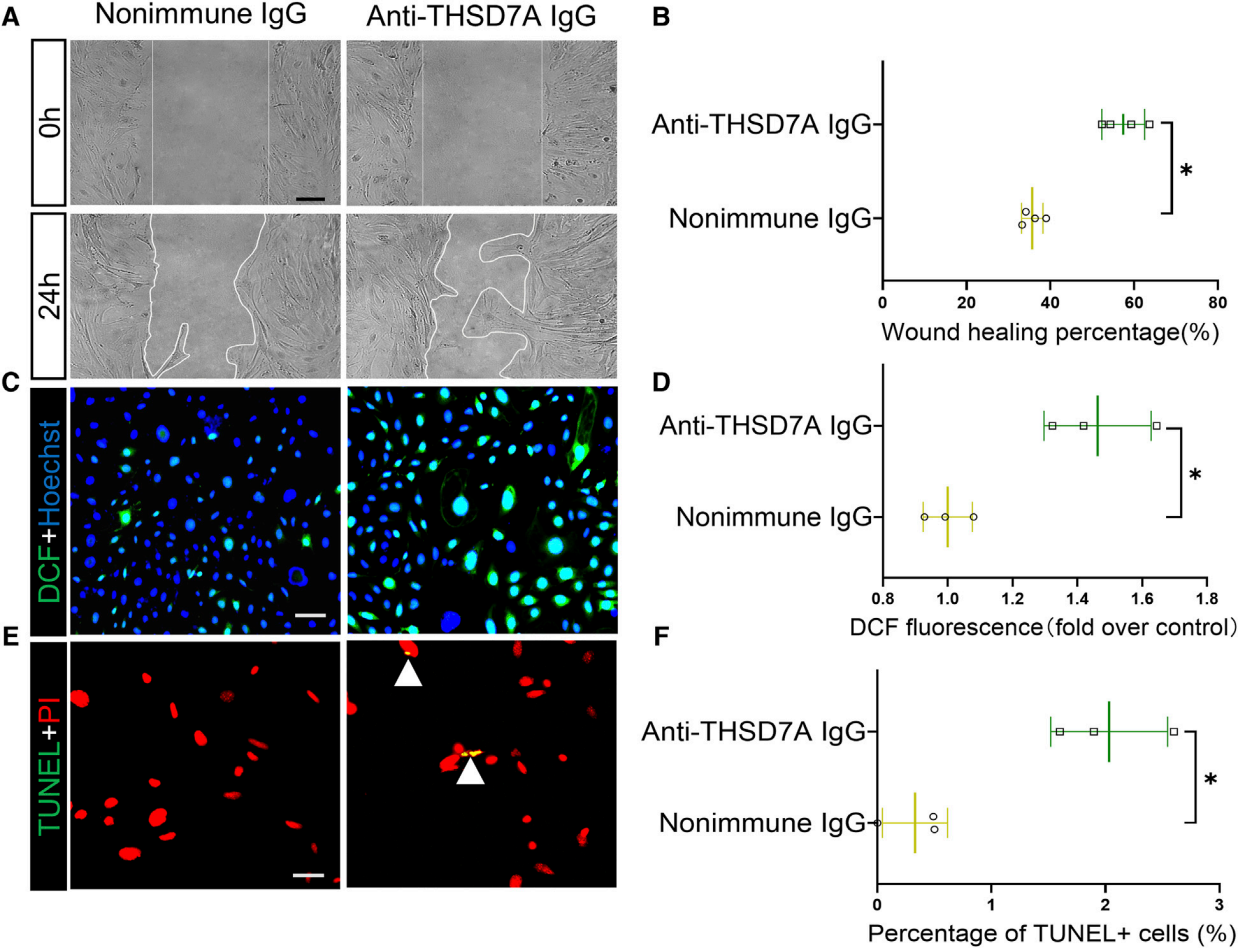
Primary podocytes exhibit hypermotility, oxidative stress and apoptosis upon exposure to the anti-THSD7A antibody. Primary cultures of mouse podocytes were exposed to the anti- THSD7A antibody at a concentration of 5 μg/mL in the presence of normal culture medium containing 10% FBS for 1 h. **(A)** Primary cultures of podocytes were treated as indicated and subsequently the scratch was processed using a 10 μL pipette. Phase-contrast micrographs were taken at 0 h and 24 h after wounding. Scale bar = 100 μm. **(B)** Cell migration areas were quantified using computerized morphometric analysis after the indicated treatments. *p < 0.05 *versus* nonimmune IgG treatment (n = 4). **(C, D)** After the indicated treatments, cells were labeled with DCF (green) for 30 min, followed by **(C)** fluorescence microscopy and **(D)** fluorometric analysis. Scale bar = 50 μm *p < 0.05 *versus* nonimmune IgG treatment (n = 3). **(E, F)** After the indicated treatments, the cells were fixed and processed for TUNEL with nuclear counterstaining for PI, followed by **(E)** dual-color fluorescence microscopy and **(F)** absolute counting of the number of TUNEL+ nuclei per high-power field. Scale bar = 50 μm *p < 0.05 *versus* nonimmune IgG treatment (n = 3).

Next, we investigated potential functional changes in podocytes induced by the anti-THSD7A antibody. Based on the cell migration assay, the anti-THSD7A antibody markedly increased podocyte motility, as shown by an accelerated closure of the gap between the leading edges of the migrating podocyte sheets after scratch wounding (Figures 3A, B). Evidence suggests that podocyte injury in MN is associated with oxidative stress (Prunotto et al., 2010). Here, after exposure to the anti-THSD7A antibody, primary podocytes indeed exhibited enhanced oxidative stress as assessed by the reactive oxygen species indicator DCF (Figures 3C, D). This was associated with increased apoptotic cellular death, as shown by TUNEL staining (Figure 3E), and by absolute counting of TUNEL-positive cells, presented as a percentage of cells per microscopic field (Figure 3F).

### Anti-THSD7A antibody targets glomerular podocytes *in vivo* and causes proteinuria in mice

**FIGURE 4:**
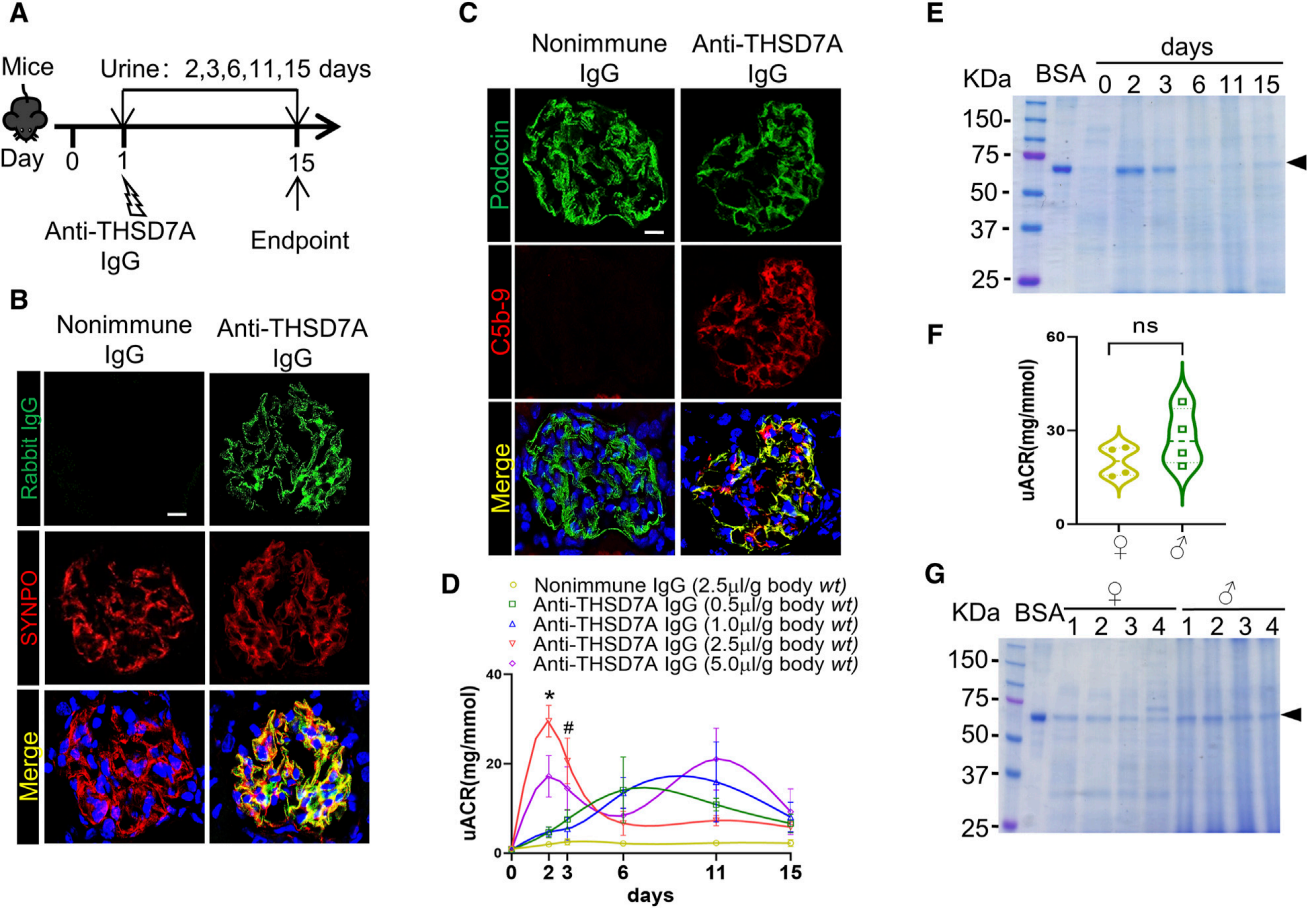
Anti-THSD7A antibody targets glomerular podocytes *in vivo* with high specificity and elicits proteinuria in mice. Mice received an intravenous injection of the anti-THSD7A antibody at a dose of 0.5, 1.0, 2.5, 5.0 μL/g body *wt*, or otherwise as indicated, and followed for 2–15 days **(A)** Schematic diagram depicts the animal experimental design. **(B, C)** Cryosections of kidney specimens from mice injured with the anti-THSD7A antibody (2.5 μL/g body *wt*) were processed for dual-color immunofluorescence staining for **(B)** rabbit IgG (green) and SYNPO (red) or **(C)** C5b-9 (red) and podocin (green) with specific antibodies. Representative fluorescent micrographs were shown (Scale bar = 10 μm). **(D)** Proteinuria was estimated using the uACR. The data was expressed as mean ± SEM and analyzed using repeated-measures ANOVA followed by *post hoc* Scheffé test. The test for a difference in uACR over time was significant (F = 3.001, *p* = 0.005). *p < 0.05 *versus* other doses, ^#^ *p* < 0.05 *versus* doses of 0.5 and 1.0 μL/g body *wt* (n = 4). **(E)** Aliquots of urine specimens from different animals injured with (2.5 μL/g body *wt*) were pooled for each time points and subjected to SDS-PAGE. **(F)** Proteinuria in female (♀) and male (♂) mice injured with (2.5 μL/g body *wt*) was estimated on day 2 using the uACR. ns, not significant (n = 4). **(G)** Representative SDS-PAGE showing urine protein on day 2 in female (♀) and male (♂) mice that were injured with (2.5 μL/g body *wt*).

To determine whether the above podocytopathic activities of this anti-THSD7A antibody observed *in vitro* could be replicated *in vivo*, mice were injected with the anti-mouse THSD7A antibody (Figure 4A). As expected from the above findings, this anti-THSD7A antibody specifically targeted glomerular podocytes with high precision, as evidenced by a perfect co-localization of the staining for rabbit IgG and SYNPO on dual-color fluorescence immunohistochemistry staining of kidney specimens (Figure 4B). This was associated with complement fixation to glomerular podocytes as evidenced by co-staining of the terminal complement complex C5b-9 with podocin (Figure 4C). As shown by uACR in Figure 4D, treatment with the anti-THSD7A antibody immediately induced massive proteinuria that peaked between day 2 to day 6 depending on the dose, followed by a gradual remission by day 15. On day 2, mice that were injected with the antibody at a dose of 2.5 μL/g body *wt* exhibited the greatest levels of albuminuria without any signs of morbidity, discomfort, or distress. Thus, the dose of 2.5 μL/g body *wt* of the anti-THSD7A antibody was chosen for subsequent experiments (Figure 4D). The uACR data were further validated by urine protein electrophoresis, which revealed that albumin was the major component of urinary protein in mice injured with this anti-THSD7A antibody (Figure 4E). In addition, no sexual disparity in albuminuria was noted in this mouse model (Figures 4F, G).

### Mice injured with the anti-THSD7A antibody develop podocytopathy and glomerular lesions characteristic of pathologic changes in human MN

**FIGURE 5:**
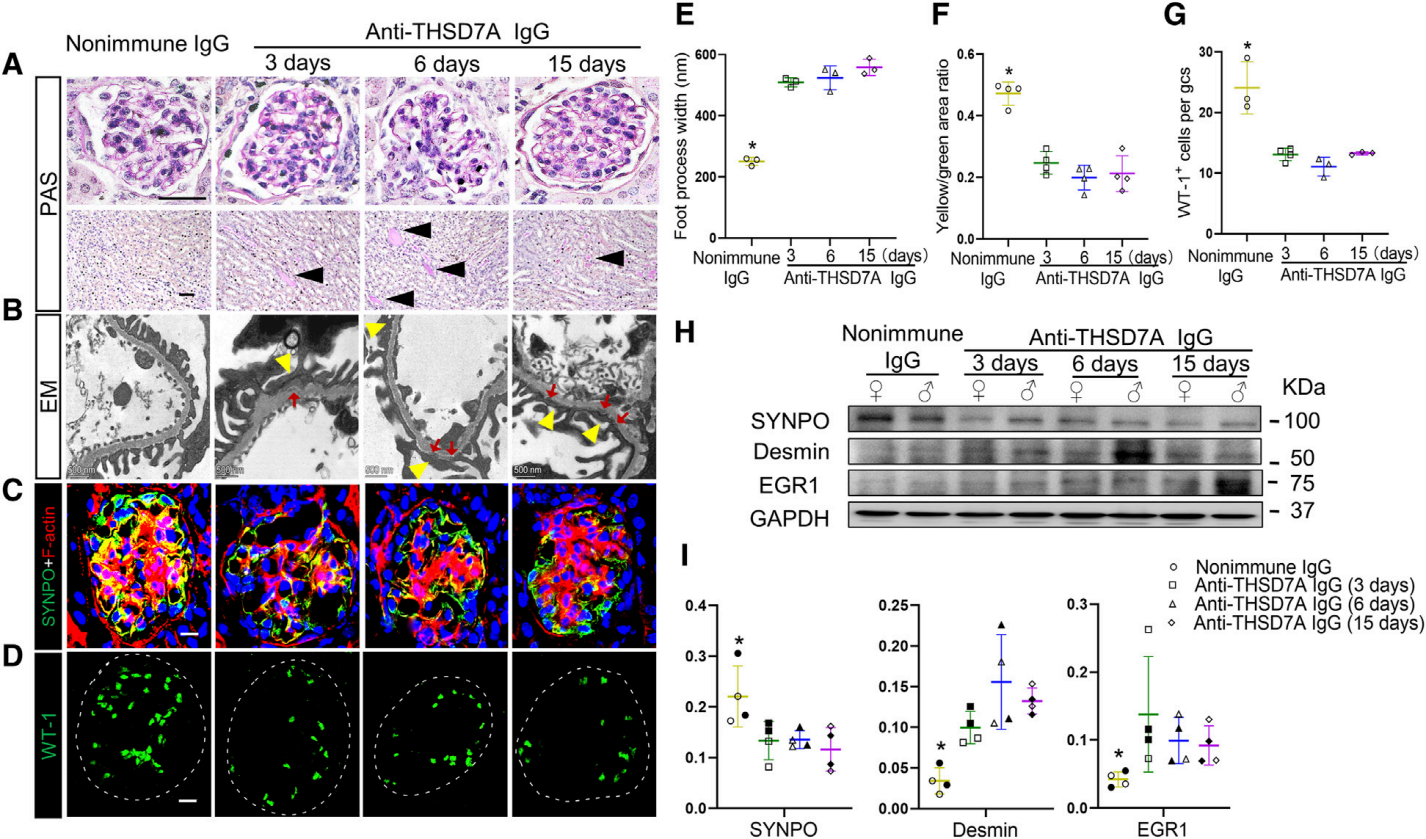
Upon the anti-THSD7A antibody insult, mice develop glomerulopathy and podocytopathy typifying pathologic changes in human MN. Mice received an intravenous injection of the anti-THSD7A antibody at a dose of 2.5 μL/g body *wt*, and were followed for indicated days **(A)** Representative micrographs of PAS staining show trivial thickening of GBM and negligible glomerular and tubulointerstitial lesions on indicated days after the anti-THSD7A antibody injury, concomitant with evident tubulointerstitial lesions and hyaline cast formation (black arrow heads) in renal tubules (scale bar = 50 µm). **(B)** Representative EM images show podocyte foot process effacement (yellow arrowheads) as well as epimembranous or intramembranous electron-dense deposits (red arrows) (scale bar = 500 nm). **(C, D)** Cryosections of kidney specimens were processed for **(C)** dual-color immunofluorescence staining for phalloidin staining of F-actin (red) and SYNPO (green) or **(D)** for WT-1 (green) with specific antibodies. Representative fluorescent micrographs were shown (Scale bar = 10 μm). **(E)** Quantification of podocyte foot process width based on **(B)**. *p < 0.05 *versus* other groups (n = 3). **(F)** Computerized morphometric analysis of the ratios of integrated pixel densities between yellow and green signals in immunofluorescence micrographs obtained in **(C)**. *p < 0.05 *versus* other groups (n = 4). **(G)** The average number of WT-1^+^ cells per glomerular cross section (gcs) was determined by absolute counting. *p < 0.05 *versus* other groups (n = 3–4). **(H)** Representative immunoblots showing immunoblot analysis of glomeruli for indicated proteins in female (♀) and male (♂) mice. **(I)** Quantification of immunoblots by densitometric analysis. The filled symbols represent males, and the empty symbols represent females. *p < 0.05 *versus* other groups (n = 4).

After anti-THSD7A antibody insult, mice showed mild glomerular lesions based on PAS staining, including trivial thickening of the GBM and negligible glomerular lesions. This was associated with evident hyaline cast formation in renal tubules and considerable tubulointerstitial lesions (Figure 5A). EM revealed epimembranous or intramembranous electron-dense deposits in glomeruli as well as variable podocyte foot process effacement in the antibody-injured group, reminiscent of glomerular ultrastructural changes in pMN (Figures 5B, E). These findings strongly suggest that a mouse model of THSD7A-associated MN has been successfully established by using this commercial antibody. Disruption of actin cytoskeleton integrity is the molecular basis for podocyte foot process effacement (Boi et al., 2023). To evaluate whether the anti-THSD7A antibody causes podocyte cytoskeletal changes, kidney specimens were subjected to both phalloidin labeling for F-actin and immunofluorescence staining for SYNPO. Confocal fluorescence microscopy demonstrated that podocyte-specific F-actin, as indicated by co-localizing signals of F-actin and SYNPO dual-color staining in glomeruli, was abundant in nonimmune IgG-treated mice, but significantly diminished after anti-THSD7A antibody injury, implying disruption of actin cytoskeleton integrity in glomerular podocytes (Figures 5C, F). This was accompanied by podocytopenia as shown by a decrease in the number of WT-1-positive podocytes in the glomeruli (Figures 5D, G). Moreover, immunoblot analysis of isolated glomeruli demonstrated that the expression of the homeostatic podocyte protein SYNPO was decreased, whereas the expression of podocyte injury markers such as desmin and EGR1 was augmented upon anti-THSD7A antibody injury. No sex discrepancy was observed in these molecular changes (Figures 5H, I). All these results suggest that this anti-THSD7A antibody-induced mouse model develops heavy proteinuria and glomerulopathy that share pathologic features with human MN.

### Both complement-dependent and -independent mechanisms contribute to the anti-THSD7A antibody-mediated glomerular injury in mice

**FIGURE 6:**
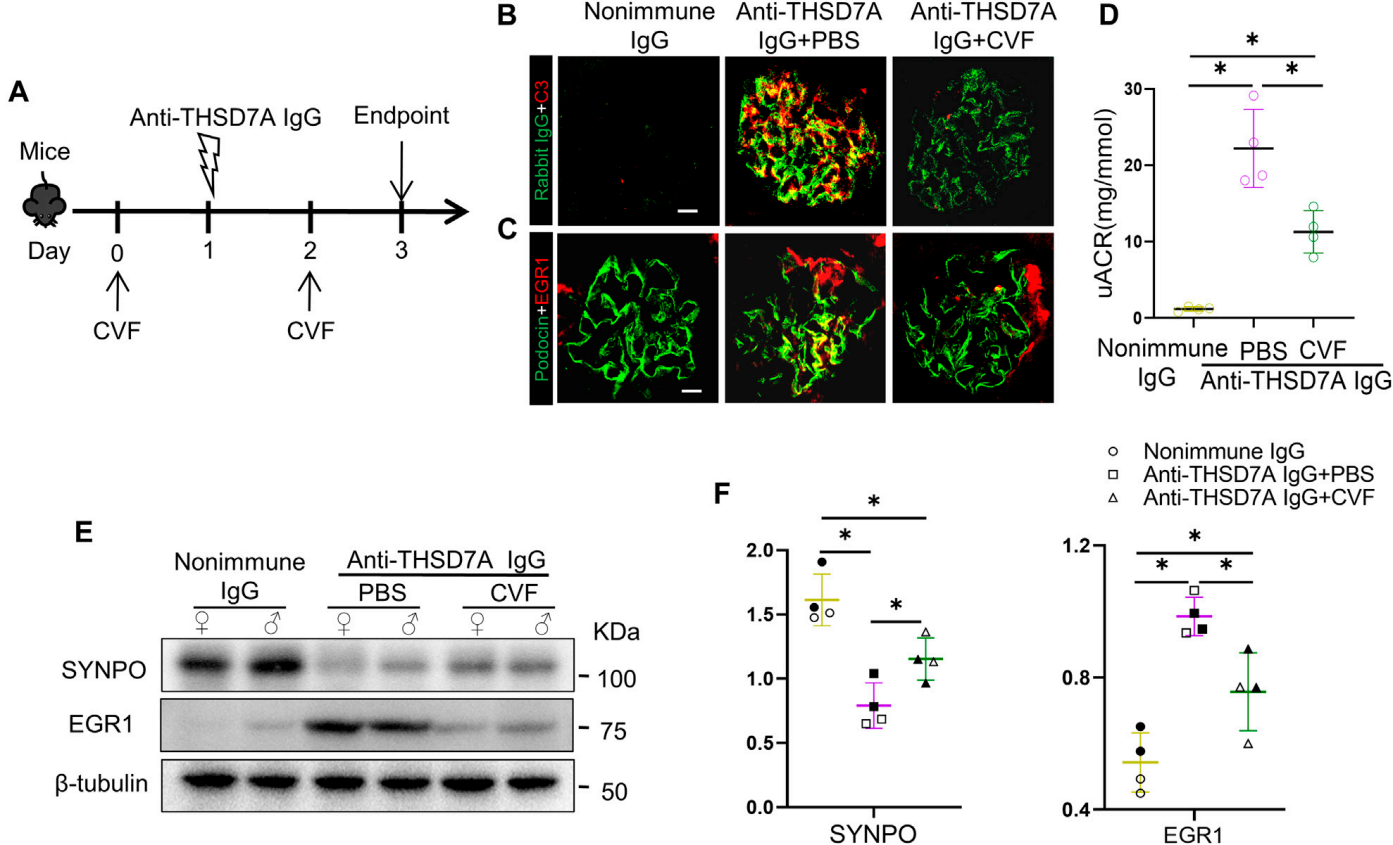
Proteinuria and glomerular injury in mice with THSD7A-associated MN are attenuated but largely preserved after complement depletion with CVF. Mice received intraperitoneal injection with 10 μg of CVF 1 day before and 1 day after the injection of the anti-THSD7A antibody (2.5 μL/g body *wt*) and were followed for 3 days **(A)** Schematic diagram depicts the animal experimental design. **(B, C)** Cryosections of kidney specimens were processed for dual-color immunofluorescence staining for **(B)** C3 (red) and rabbit IgG (green), or for **(C)** EGR1 (red) and podocin (green) with specific antibodies. Representative fluorescent micrographs were shown (Scale bar = 10 μm). **(D)** Proteinuria was estimated using the uACR. *p < 0.05 *versus* other groups (n = 4). **(E)** Representative immunoblots showing immunoblot analysis of glomeruli for indicated proteins in female (♀) and male (♂) mice. **(F)** Quantification of immunoblots by densitometric analysis. The filled symbols represent males, and the empty symbols represent females. *p < 0.05 *versus* other groups (n = 4).

To determine the role of complements in this mouse model of THSD7A-associated MN, mice received intraperitoneal injection with CVF prior to anti-THSD7A antibody injury (Figure 6A). Consistent with its complement-depleting property, CVF injection abolished glomerular C3 deposition in mice injured with the anti-THSD7A antibody, denoting a successful complement depletion (Figure 6B). This was associated with a partial attenuation of podocyte injury, marked by reduced expression of podocyte-specific EGR1 based on dual-color fluorescence immunohistochemistry staining for EGR1 and podocin (Figure 6C). In parallel, complement depletion with CVF also attenuated albuminuria in mice injured with anti-THSD7A antibody. Meanwhile, immunoblot analysis of isolated glomeruli showed that CVF pre-treatment mitigated the loss of SYNPO and the induction of EGR1 in glomeruli upon the anti-THSD7A antibody injury. However, despite CVF pre-treatment, anti-THSD7A antibody was still able to elicit considerable proteinuria and podocyte injury, though to a lesser extent (Figures 6D–F). In addition, no sex disparity in the expressions of SYNPO and EGR1 were noted in mice that were injured with anti-THSD7A antibody after CVF pre-treatment (Figures 6E, F). These findings suggest that complement-mediated glomerular injury is involved in this mouse model of THSD7A-associated MN, but other complement-independent pathomechanisms may also contribute.

### Anti-THSD7A antibody-mediated podocytopathy involves both complement-dependent mechanism and autonomous podocyte injury

**FIGURE 7:**
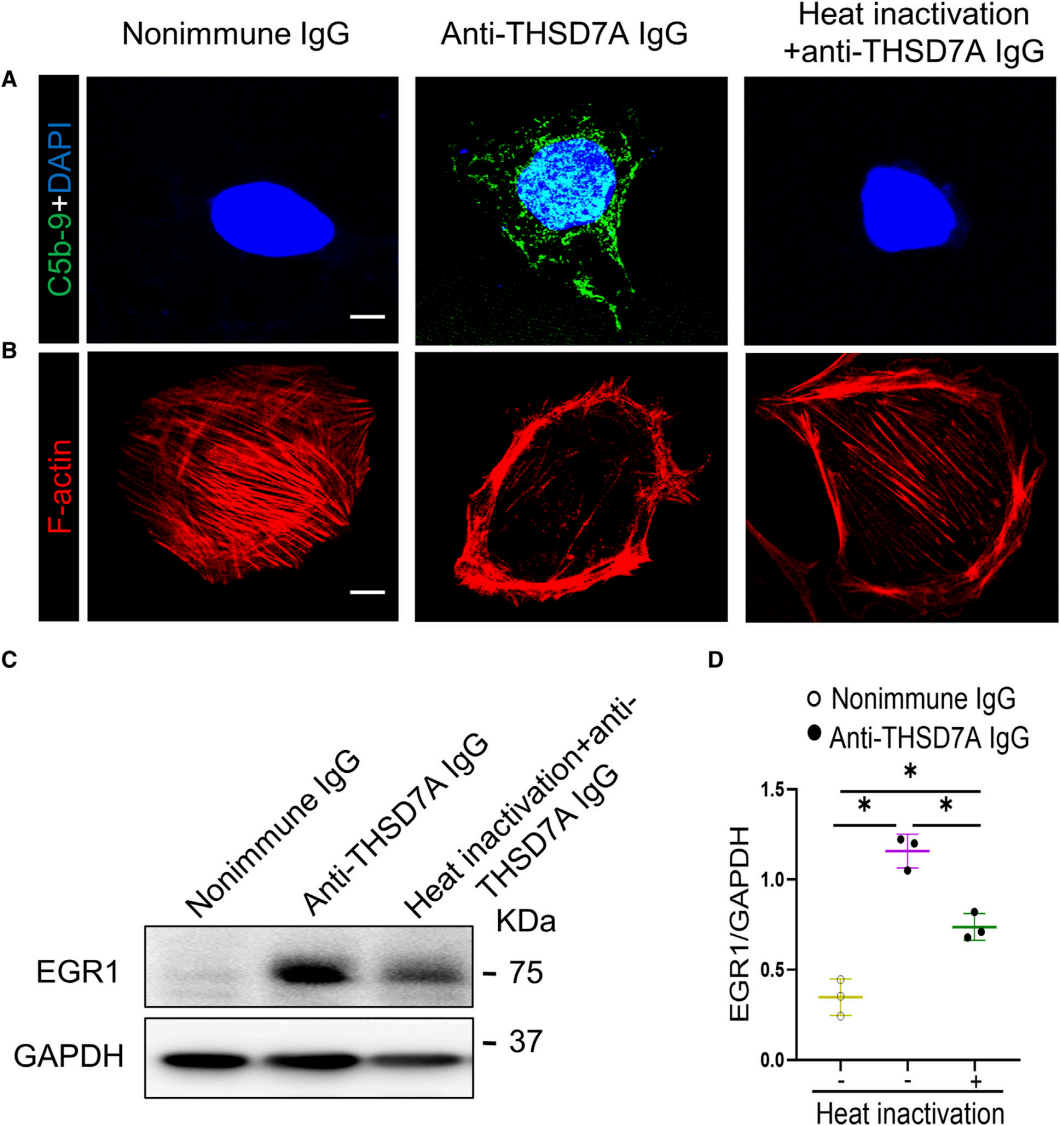
Both complement-dependent and -independent mechanisms are implicated in the anti-THSD7A antibody-elicited podocyte injury. Primary cultures of mouse podocytes were exposed to the anti-THSD7A antibody at a concentration of 5 μg/mL in the presence of normal culture medium containing 10% FBS or heat-inactivated culture medium for 1 h. **(A, B)** Representative micrographs showing fluorescent staining for **(A)** C5b-9 (green) and **(B)** F-actin (red) in primary mouse podocytes. Scale bar = 10 μm. **(C)** Representative immunoblot analysis of cell lysates for EGR1. **(D)** Quantification of the immunoblot data by densitometric analysis. *p < 0.05 (n = 3).

To validate whether both complement-dependent and -independent mechanisms are responsible for podocytopathy elicited by the anti-THSD7A antibody, primary podocytes were cultured in normal complete medium containing complements or in medium containing heat-inactivated FBS, which inactivates heat-labile complements (Figure 7A). Addition of the anti-THSD7A antibody to podocytes cultured in complete medium caused drastic disruption of actin cytoskeleton, as assessed by phalloidin staining. This deleterious effect was partially abolished in podocytes cultured in complement-inactivated medium, but was still largely preserved (Figure 7B). The morphologic findings were further corroborated by immunoblot analysis coupled with densitometric analysis, which demonstrated that the inducing effect of the anti-THSD7A antibody on the podocyte injury marker EGR1 was partially diminished, but still largely retained in podocytes cultured in complement-inactivated medium (Figures 7C, D). These results suggest that in addition to complement-dependent cytotoxicity, autonomous podocyte injury mediated by a direct attack of the anti-THSD7A antibody may also be involved in this model of THSD7A-related podocytopathy.

### RCI improves proteinuria and glomerular injury in this mouse model of THSD7A-associated MN

**FIGURE 8:**
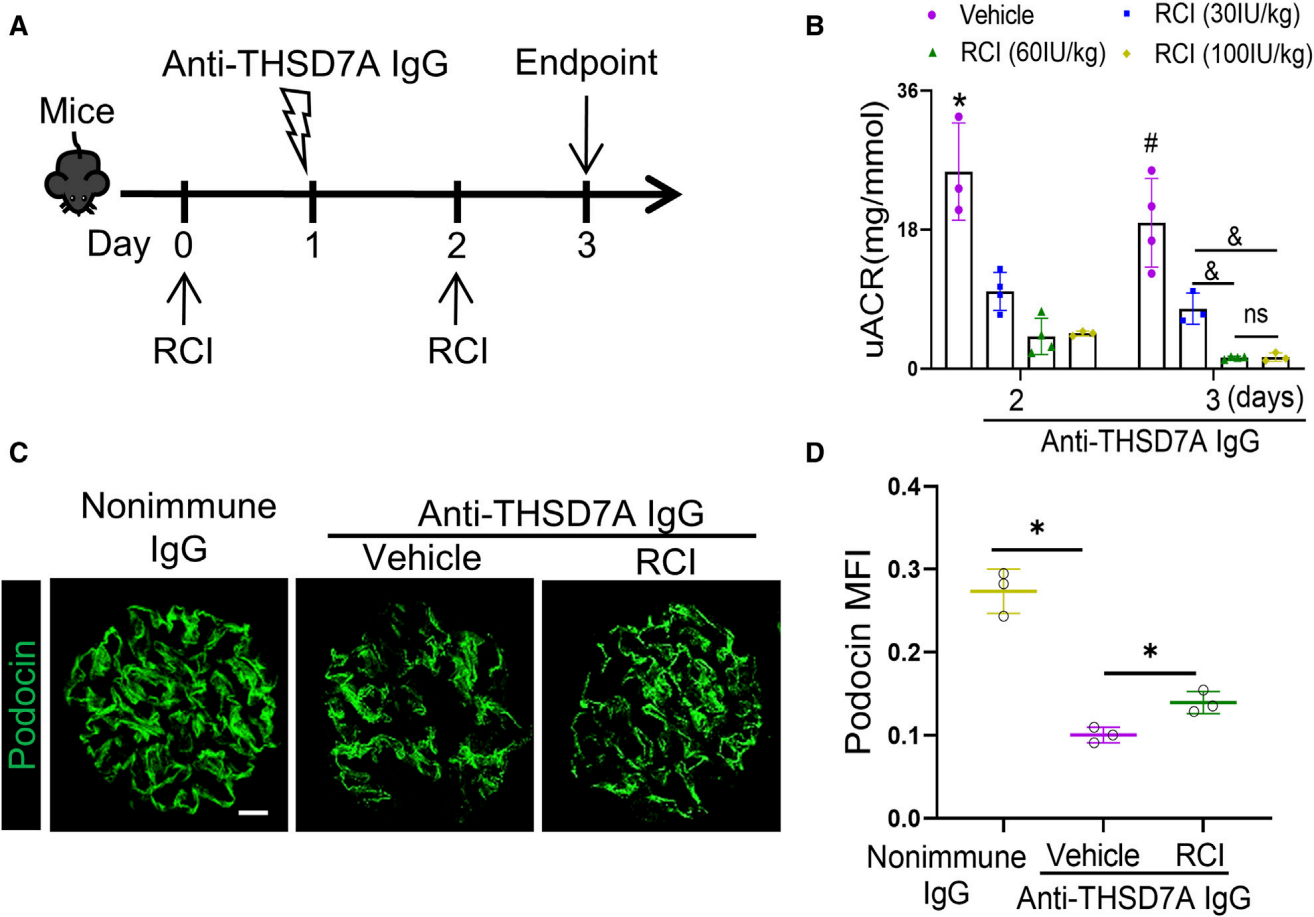
ACTH therapy with RCI ameliorates proteinuria and glomerular injury in mice with THSD7A-associated MN. Mice received subcutaneous RCI injection every other day at varying doses starting 1 day before anti-THSD7A antibody (2.5 μL/g body *wt*) insult and were followed for 3 days. **(A)** Schematic diagram depicts the animal experimental design. **(B)** Proteinuria was estimated using the uACR. *p < 0.05 *versus* other groups on day 2; ^#^ *p* < 0.05 *versus* other groups on day 3; ^&^ *p* < 0.05. ns, not significant (n = 3–4). **(C)** Cryosections of kidney specimens were processed for immunofluorescence staining for podocin (green). Scale bar = 10 μm. **(D)** Computerized morphometric analysis of mean fluorescence intensity (MFI) of podocin staining in glomeruli. *p < 0.05 (n = 3).

To test the utility of this mouse model in the development of treatments for MN, adrenocorticotropic hormone (ACTH) therapy with RCI, the first FDA-approved treatment for pMN, was administered to this mouse model at varying doses every other day starting 1 day before the anti-THSD7A antibody insult (Figure 8A). Shown by the uACR data in Figure 8B, RCI effectively attenuated proteinuria in a dose-dependent manner on day 2 and day 3, the proteinuria-lowering effect of RCI appears to plateau above the dose of 60IU/kg body *wt* (Figure 8B). This anti-proteinuric effect of RCI was associated with an improvement in glomerular injury, as evidenced by more preserved staining for the podocyte canonical marker protein podocin on fluorescence immunohistochemistry staining of kidney specimens (Figures 8C, D). Collectively, these data indicate a renoprotective effect of RCI in experimental THSD7A-associated MN.

## Discussion

**FIGURE 9:**
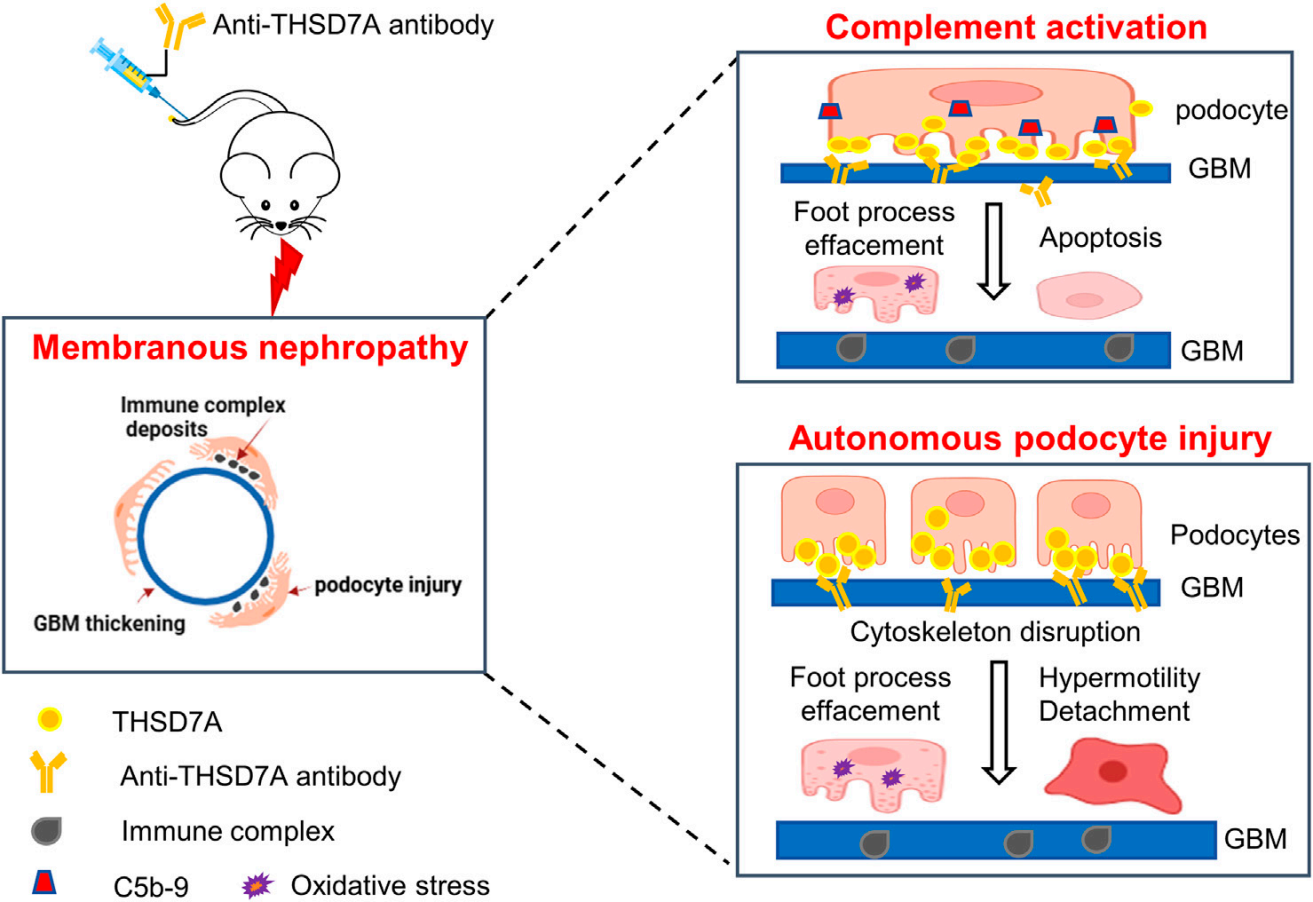
Schematic representation of the working model for the pathomechanisms involved in the THSD7A-associated MN. In this study, a mouse model of THSD7A-associated MN was developed by administration of a commercially available antibody targeting the most N-terminal part of mouse THSD7A. The underlying pathomechanims seem to be twofold. On one hand, as a typical immune-complex mediated disease, this model of THSD7A-associated MN involves complement-mediated podocyte injury, characterized by podocyte foot process effacement, podocyte oxidative stress and apoptosis, and GBM thickening. In addition, the anti-THSD7A antibody may lead to reduced activity of THSD7A, which has been shown to cause disruption of actin cytoskeleton integrity, possibly resulting in an autonomous podocyte injury, marked by podocyte foot process effacement, podocyte hypermobility, and podocyte detachment. Both the complement-dependent and -independent podocytopathic mechanisms contribute to anti-THSD7A antibody-elicited glomerular injury, which recapitulates human THSD7A-associated MN in its hisopathologic changes and severe proteinuria.

By using a commercially available anti-THSD7A antibody, this study developed a heterologous mouse model of THSD7A-associated MN. Compared with a previous model using a proprietary in-house antibody (Tomas et al., 2016; Tomas et al., 2017), our model, based on the use of a commercially available antibody targeting the most N-terminal part of THSD7A, would be instrumental for broad application in MN research, including the evaluation of new treatments, as exemplified here by testing the RCI therapy. No sex difference was observed in our model of THSD7A-associated MN. Importantly, our study demonstrated that THSD7A-associated MN, at least in this model, involves both complement-dependent and independent pathogenic mechanisms (Figure 9).

As a quintessential immune complex-mediated glomerular disease, complement-dependent injurious mechanisms have been centrally implicated in the pathogenesis of MN (Chen et al., 2023; Kistler and Salant, 2024). Indeed, biopsy-proven MN in human patients is always associated with complement fixation and deposition of MAC in diseased glomeruli (Ayoub et al., 2021; Seifert et al., 2023). In experimental MN such as active Heymann nephritis (AHN) and PHN, sublytic glomerular epithelial cell injury mediated by MAC leads to abnormal GBM matrix formation and typical GBM thickening (Takano and Cybulsky, 2000; Pippin et al., 2003). However, this conventional dogma about complement-mediated injury and MN has been challenged by new evidence. For example, in human patients with pMN, the dominant subclass of MN-associated autoantibodies, such as anti-PLA2R1 and anti-THSD7A autoantibodies, is IgG4 (Kaya, Paydas, Balal, Eren Erdogan and Gonlusen, 2021), which is known to be a weak activator of complements due to its low affinity to C1q and Fcγ receptors (Vidarsson, Dekkers and Rispens, 2014), although it has been reported that underglycosylation of IgG4 may promote the activation of the lectin complement pathway in MN (Hayashi et al., 2018; Z. Wang, Wen, Dou and Zhao, 2018; Zhang et al., 2020), and that IgG4-dominant anti-THSD7A autoantibodies can activate the alternative pathway in an *in vitro* complement activation assay (Manral, Caza, Storey, Beck and Borza, 2022). More importantly, in a multicenter, double-blind, placebo-controlled trial in pMN, eculizumab, a humanized inhibitory anti-C5 monoclonal antibody, showed no difference in proteinuria compared to the placebo group after 16 weeks of treatment (Thomas et al., 1996). Similarly, in experimental MN such as AHN and PHN, glomerular injury or proteinuria was not affected by C6 knockout and the ensuing MAC deficiency (Leenaerts, Hall, Van Damme, Daha and Vanrenterghem, 1995). This directly contradicts previous observations that complement depletion with CVF prevents proteinuria in PHN during the heterologous or autologous phase (Perkinson, Baker, Couser, Johnson and Adler, 1985).

The reason for the above controversial findings is unknown but it is possible that complement-independent mechanisms are also at play in pMN. In support of this contention, passive transfer of human anti-THSD7A to mice elicited proteinuria before any detection of complement activation (Tomas et al., 2016). Also in the present study, using our mouse model of THSD7A-associated MN, CVF was found to only partially attenuate proteinuria and glomerular injury. Furthermore, *in vitro* in podocytes cultured in medium containing heat-inactivated FBS in which heat-labile complements have been inactivated, the nephritogenic anti-THSD7A antibody is still able to cause podocyte injury, albeit to a lesser extent. The mechanisms responsible for the complement-independent podocytopathy are currently unknown and may depend on the specific autoantigens involved. Renal expression of THSD7A is highly enriched in podocytes, but its role in podocyte pathobiology has not yet been defined. Nevertheless, studies in human vascular endothelial cells have shown that THSD7A co-localizes with αv/β3-integrin and paxillin at the leading edge of migrating cells and may play a role in regulating cytoskeletal organization and cellular dynamics (C. H. Wang et al., 2010). Indeed, inhibition of THSD7A by RNA interference increased cell motility (Liu et al., 2016). Consistently, in cultured human podocytes, THSD7A localizes to thin arborized protrusions and filopodia, and THSD7A expression dampens the activity of cytoskeletal regulatory proteins, increases podocyte adhesion and impedes motility. *In vivo* in renal glomeruli, THSD7A localizes to podocyte foot processes in close proximity to the slit diaphragm, suggesting that THSD7A may be involved in stabilizing the slit diaphragm (Herwig et al., 2019). The anti-THSD7A autoantibodies prepared from pMN patients as well as the rabbit anti-THSD7A antibody used to develop the experimental MN appear to exert an inhibitory effect on THSD7A, as these antibodies, when applied to cultured podocytes (Tomas et al., 2016), caused cytoskeletal rearrangement, disrupted podocyte cytoskeletal architecture and activation of focal adhesion signaling. These changes are consistent with podocyte hypermobility. However, it cannot be excluded that the observed changes in podocyte cytoskeletons in the above studies were due to antibody-activated complements, because podocytes were cultured in medium supplemented with FBS that may contain complements (Tomas et al., 2016; Tomas et al., 2017). Therefore, it is still uncertain whether the anti-THSD7A antibody has a direct podocytopathic activity. To address this issue, we exposed podocytes to the anti-THSD7A antibody in a complement-inactivated culture medium. In the absence of complements, the anti-THSD7A antibody was still able to cause autonomous podocyte injury (Figure 7), characterized by disruption of the actin cytoskeleton integrity and increased expression of the podocyte injury marker EGR1. Podocyte injury induced by the anti-THSD7A antibody was further exacerbated when cells were cultured in complete medium, suggesting a dual podocytopathic effect of the anti-THSD7A antibody, namely, complement-mediated injury and podocyte autonomous injury.

It could be argued that either CVF treatment *in vivo* or heat inactivation of FBS *in vitro* may not completely deplete the complements, and thus the residual complement activity may account for the preserved podocytopathic effects of the anti-THSD7A antibody. To address this concern, we also performed a short-term cell culture experiment of podocytes in serum-free medium, and the podocytopathic effects of the anti-THSD7A antibody were also observed (data not shown), indicating autonomous podocyte injury. Nevertheless, the complement-independent glomerulopathic effect of the anti-THSD7A antibody merits further examination in animals lacking key complement components in the future.

In summary, we have successfully established a mouse model of THSD7A-associated MN by administration of a commercially available antibody against THSD7A. Our study demonstrated that the pathogenesis of THSD7A-associated MN involves both complement-dependent podocyte injury and autonomous podocytopathy (Figure 9). ACTH treatment with the FDA-approved RCI appears to be beneficial in this model, although the therapeutic mechanism warrants further investigation. This model, which utilizes the commercially available anti-THSD7A antibody, will be an important tool for the research community to explore the pathogenesis and therapeutic modalities of MN.

## Data Availability

The original contributions presented in the study are included in the article/Supplementary Material, further inquiries can be directed to the corresponding author.
