# Supplementary material for: THSD7A-associated membranous nephropathy involves both complement-mediated and autonomous podocyte injury
Source: Front Pharmacol. 2024 Jul 17;15:1430451. doi: 10.3389/fphar.2024.1430451 (PMC11288966; doi:10.3389/fphar.2024.1430451)
Supplement: Supplementary file 1 [file Table1.DOC]

**Supplementary table 1.** Detailed information of antibodies used in this study.

| **Antibody** | **Company** | **Catalogue NO.** |
| --- | --- | --- |
| THSD7A | LS Bio | [LS-C315036](https://www.lsbio.com/antibodies/thsd7a-antibody-aa42-242-wb-western-ls-c315036/325206) |
| Podocin | Santa Cruz Biotechnology | SC-22298 |
| Synaptopodin | Santa Cruz Biotechnology | SC-515842 |
| Desmin | Santa Cruz Biotechnology | SC-7559 |
| WT-1 | Santa Cruz Biotechnology | SC-15421 |
| C5b-9 | Santa Cruz Biotechnology | SC-66190 |
| C3 | Santa Cruz Biotechnology | SC-28294 |
| GAPDH | Santa Cruz Biotechnology | SC-32233 |
| EGR1 | Thermo Fisher | H00001958-M03 |
| β-tubulin | Cell Signaling Technology | 2128S |
| Mouse anti-rabbit IgG-HRP | Santa Cruz Biotechnology | SC-2357 |
| Mouse anti-goat IgG-HRP | Santa Cruz Biotechnology | SC-2354 |
| Goat anti-mouse IgG-HRP | Invitrogen | 31430 |
| F-actin (Alexa Fluor 594 phalloidin) | Invitrogen | A12381 |
| Alexa Fluor 488 goat anti-rabbit IgG (H+L) | Invitrogen | A11008 |
| Alexa Fluor 594 chicken anti-mouse IgG (H+L) | Invitrogen | A21201 |
| Alexa Fluor 488 chicken anti-mouse IgG (H+L) | Invitrogen | A21200 |
| Alexa Fluor 488 donkey anti-goat IgG (H+L) | Invitrogen | A11055 |
| Alexa Fluor 594 donkey anti-rabbit IgG (H+L) | Invitrogen | A21207 |
