# Supplementary material for: THSD7A-associated membranous nephropathy involves both complement-mediated and autonomous podocyte injury
Source: Front Pharmacol. 2024 Jul 17;15:1430451. doi: 10.3389/fphar.2024.1430451 (PMC11288966; doi:10.3389/fphar.2024.1430451)
Supplement: Supplementary file 2 [file DataSheet1.PDF]

Supplementary Information:

THSD7A-associated membranous nephropathy involves both complement-mediated and autonomous podocyte injury

Jing Liu, Yan Ge, William Gunning, Lance Dworkin, Rujun Gong

Division of Nephrology, the University of Toledo College of Medicine, Toledo, Ohio, USA.

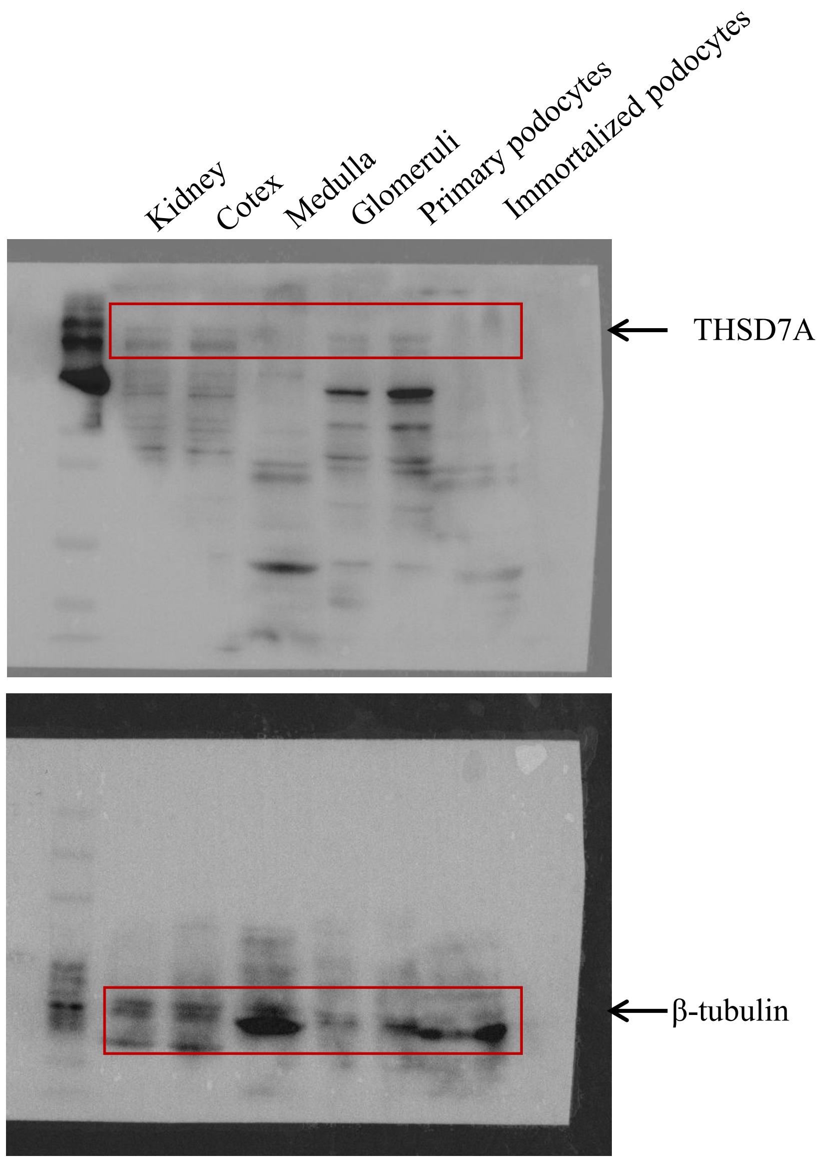

Supplementary Figure 1: Uncropped blots used for the construction of composite of Figure 1.

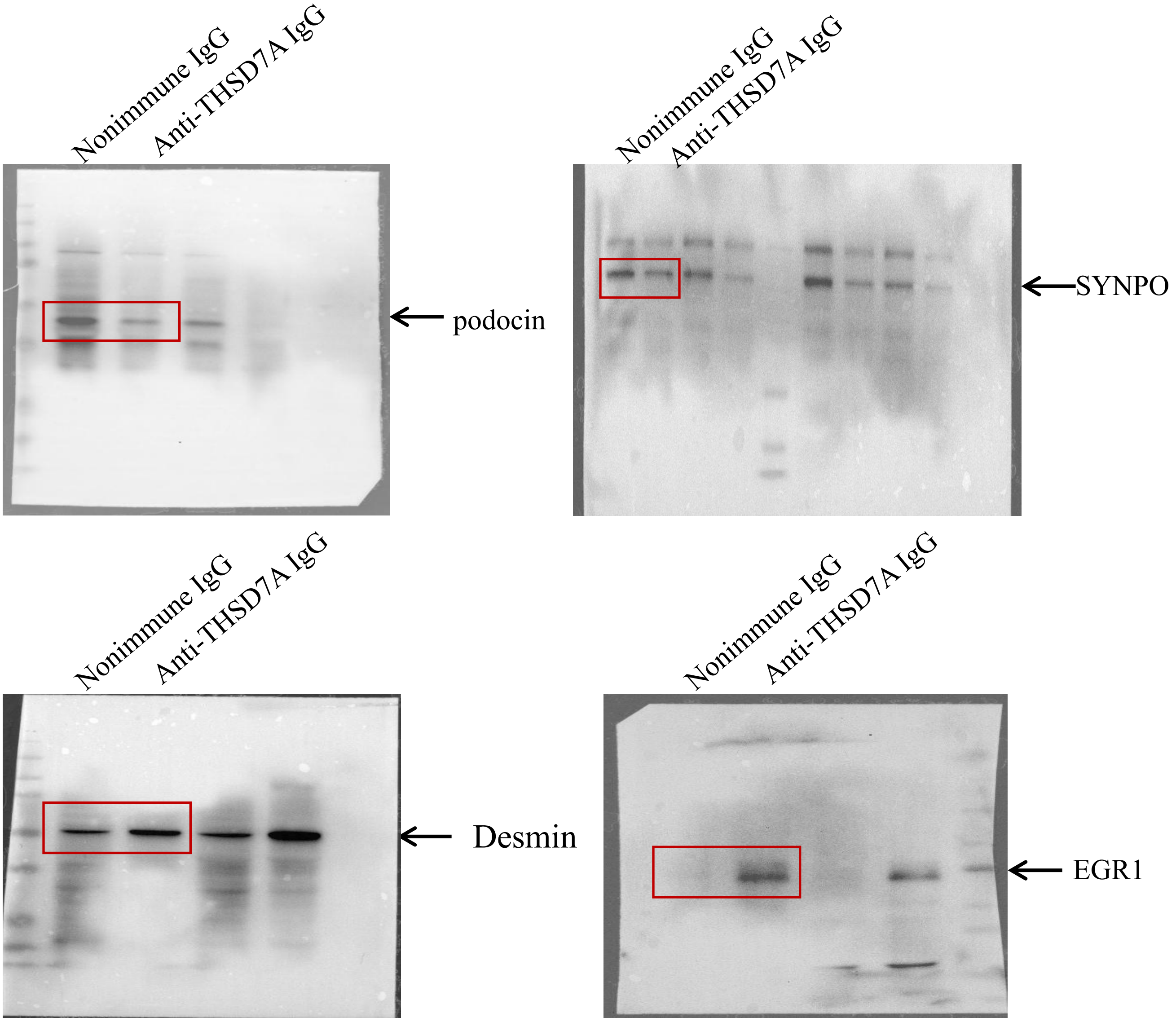

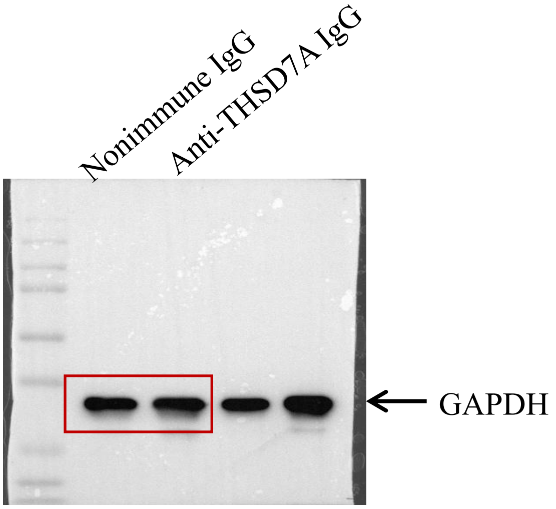

Supplementary Figure 2: Uncropped blots used for the construction of composite of Figure 2.

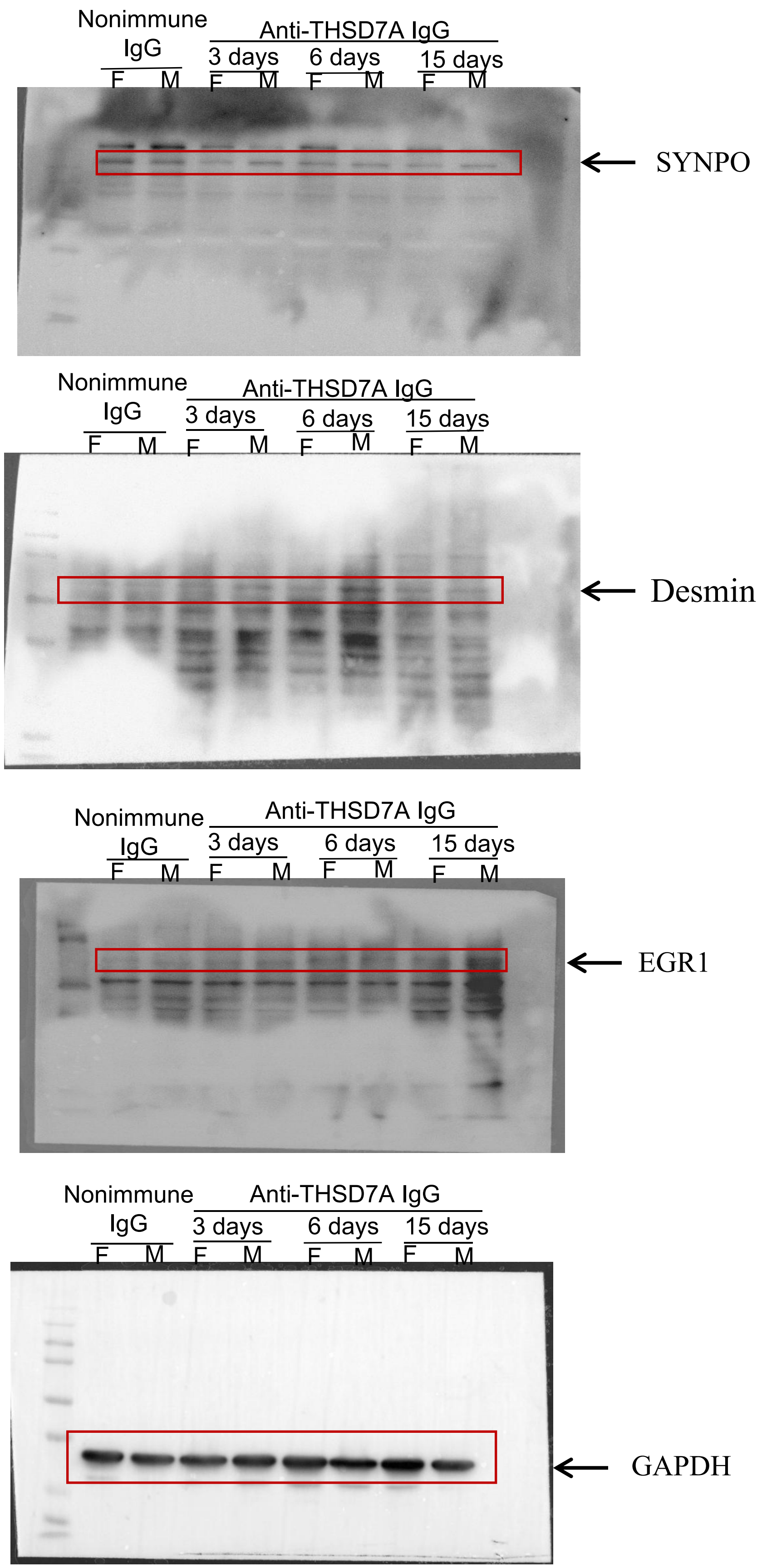

Supplementary Figure 3: Uncropped blots used for the construction of composite of Figure 5.

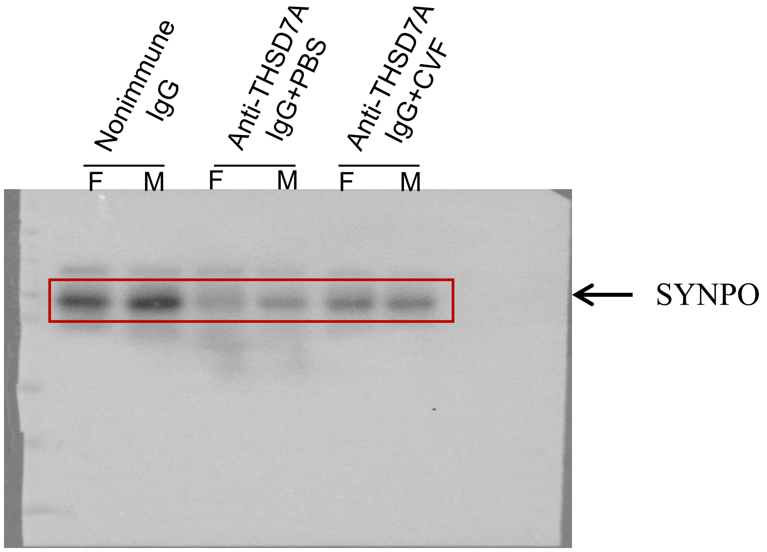

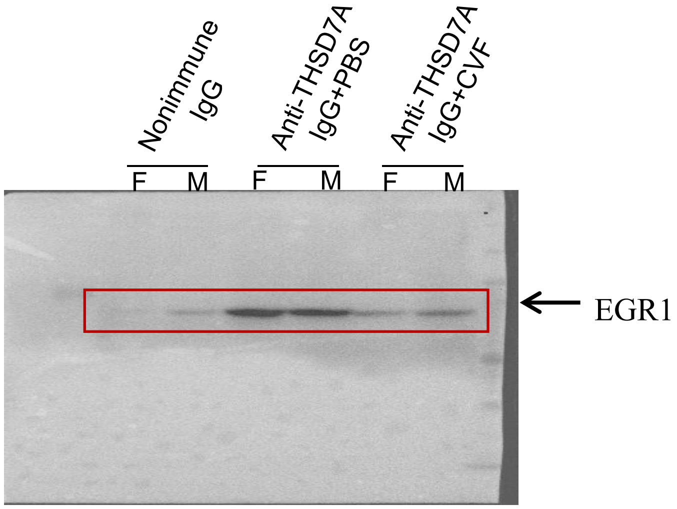

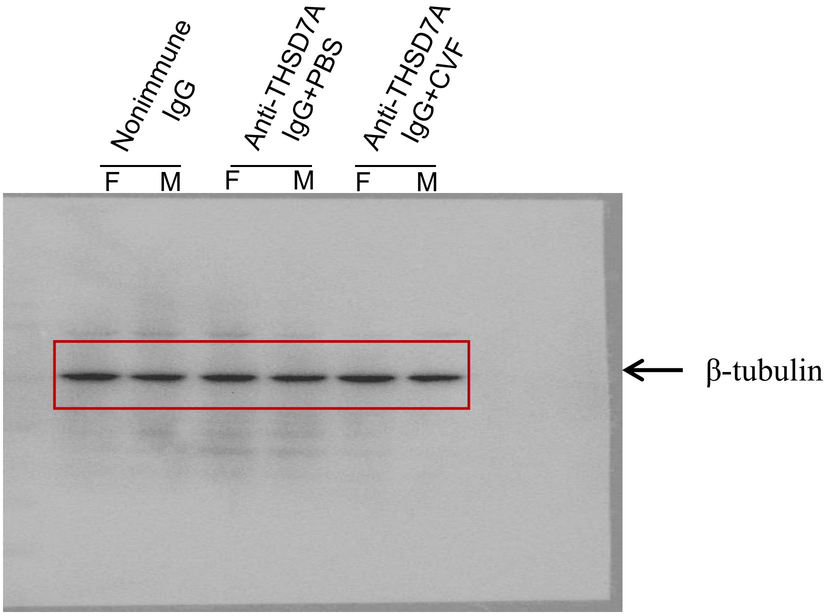

Supplementary Figure 4: Uncropped blots used for the construction of composite of Figure 6.

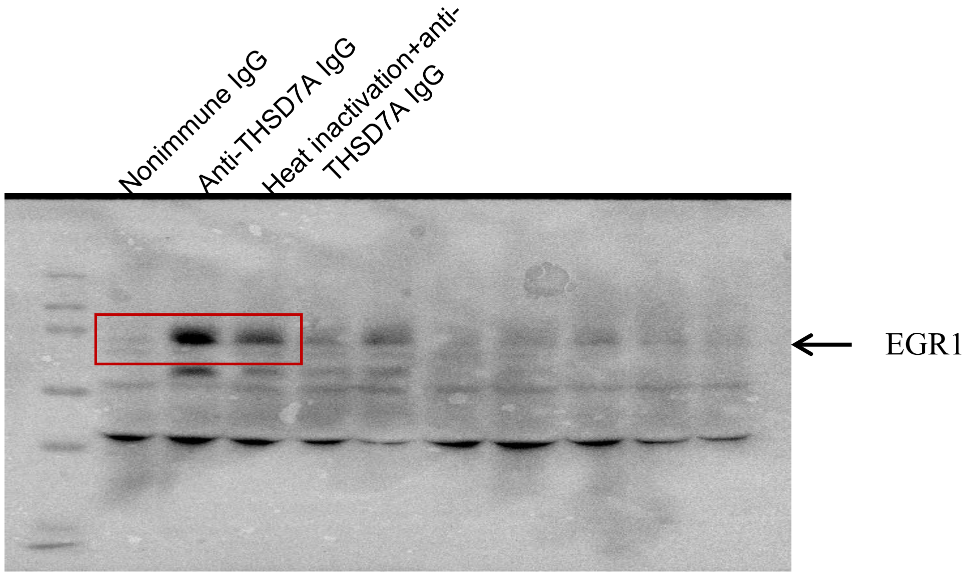

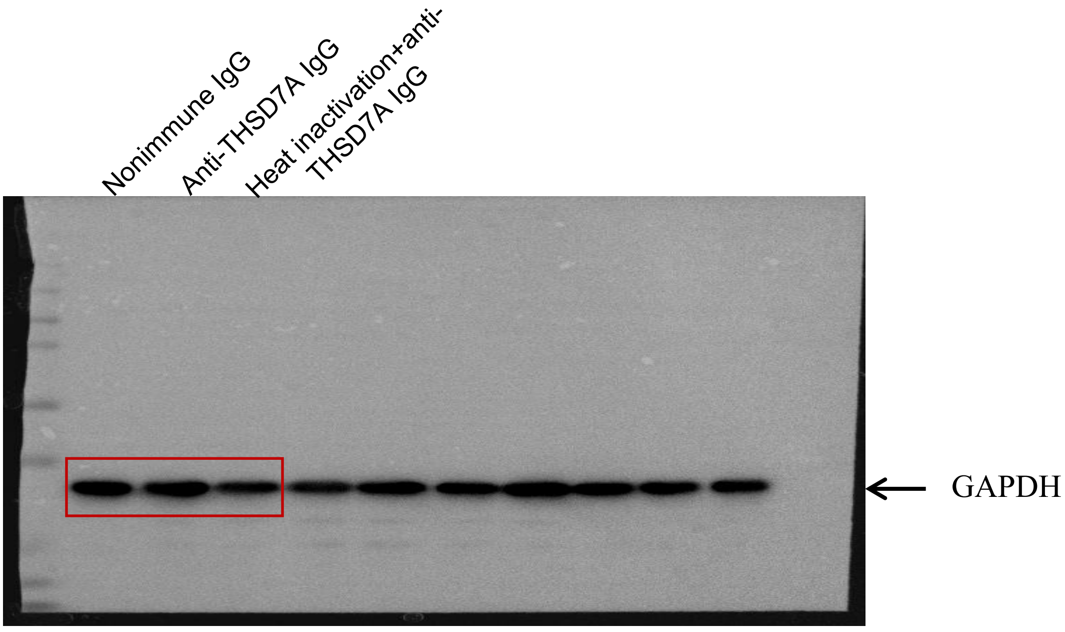

Supplementary Figure 5: Uncropped blots used for the construction of composite of Figure 7.
